# Solution-Crystallization and Related Phenomena in 9,9-Dialkyl-Fluorene Polymers. I. Crystalline Polymer-Solvent Compound Formation for Poly(9,9-dioctylfluorene)

**DOI:** 10.1002/polb.23798

**Published:** 2015-08-19

**Authors:** Aleksandr Perevedentsev, Paul N Stavrinou, Donal D C Bradley, Paul Smith

**Affiliations:** 1 Department of Physics and Centre for Plastic Electronics, Imperial College LondonLondon, SW7 2AZ, United Kingdom; 2 Department of Materials, Eidgenössische Technische Hochschule (ETH) ZürichVladimir-Prelog-Weg 5, 8093, Zürich, Switzerland

**Keywords:** conjugated polymers, crystallization, microstructure

## Abstract

Polymer-solvent compound formation, occurring via co-crystallization of polymer chains and selected small-molecular species, is demonstrated for the conjugated polymer poly(9,9-dioctylfluorene) (PFO) and a range of organic solvents. The resulting crystallization and gelation processes in PFO solutions are studied by differential scanning calorimetry, with X-ray diffraction providing additional information on the resulting microstructure. It is shown that PFO-solvent compounds comprise an ultra-regular molecular-level arrangement of the semiconducting polymer host and small-molecular solvent guest. Crystals form following adoption of the planar-zigzag β-phase chain conformation, which, due to its geometry, creates periodic cavities that accommodate the ordered inclusion of solvent molecules of matching volume. The findings are formalized in terms of nonequilibrium temperature–composition phase diagrams. The potential applications of these compounds and the new functionalities that they might enable are also discussed. © 2015 The Authors. Journal of Polymer Science Part B: Polymer Physics published by Wiley Periodicals, Inc. J. Polym. Sci., Part B: Polym. Phys. **2015**, *53*, 1481–1491

## INTRODUCTION

Poly(9,9-dioctylfluorene) (PFO) is a widely studied conjugated polymer that exhibits a broad range of desirable properties, such as efficient pure-blue photo (PL) and electroluminescence (EL), high charge–carrier mobility and optical gain, good thermal stability and excellent processability from solutions in common organic solvents.^1^–^3^ It has further been shown that solid-state PFO can be controllably fabricated in a range of glassy, liquid- and semi-crystalline microstructures that directly influence the resulting optoelectronic properties.^4^–^11^ These features make PFO well-suited for applications in a variety of optoelectronic devices, such as LEDs,^12^ lasers,^3^,^13^ sensors,^14^,^15^ and photonic elements,^16^–^18^ while also allowing it to be used as a test-bed for fundamental studies of conjugated polymer photophysics.^3^,^7^,^19^ The 9,9-dioctylfluorene unit is additionally found in a wide variety of copolymer structures.^20^–^22^

Solution-crystallization of PFO, typically manifested as thermoreversible gelation when solution concentration is sufficiently high for substantial chain overlap,^23^ has been demonstrated for a range of organic solvents, such as cyclohexane,^5^,^6^ α-pinene,^6^ dichloroethane,^24^ methylcyclohexane (MCH),^25^–^27^ and toluene.^28^ In an early study Grell et al.^6^ tentatively excluded polymer-solvent compound formation as a contributory factor due to the literature teaching that such process should occur only for good solvents;^29^ this turns out not to be the case (*vide infra*). The fundamentally intra-chain nature of the processes responsible for the observed changes in absorption and PL spectra was nevertheless recognized, with the distinct planar-zigzag β-phase chain segment conformation shown to be a common feature of PFO–cyclohexane gels, vapor-annealed or thermally-treated PFO and PFO:polystyrene dilute blend films, and of vapor-annealed PFO quenched nematic glass fibres.^6^ Later studies used a combination of light, X-ray and neutron scattering as well as optical spectroscopy to further investigate the photophysical and structural properties of solution-crystallized PFO. In MCH and toluene PFO was found to crystallize into sheet-like structures, usually referred to as “aggregates,” with lateral dimensions on the order of 10–100 nm and thickness of a few nm.^25^–^28^,^30^ Subsequent agglomeration of these sheets into ribbon-like structures allowed thermoreversible crosslinking and gelation^24^,^31^ but the sheet-like structures themselves invariably displayed the spectroscopic and crystallographic signatures of β-phase chain segments.^26^,^28^ Despite the results of these previous studies, solution-crystallization of PFO has been rather poorly understood due to several fundamental ambiguities. First, the number and nature of the phase-transitions that result in the formation of crystalline structures in PFO solutions remains unclear. Second, although both intra- and inter-chain structure formation, mediated by β-phase chain segment and sheet-like domain generation, respectively, has been observed upon solution crystallization, the relationship between these phenomena has not been unequivocally determined.

This confused situation motivated our present study and we report below on the formation and selected properties of solution-crystallized polymer-solvent compounds, comprising PFO and particular organic solvents. Such compounds form by stoichiometric co-crystallization of polymer chains and small-molecular solvents, resulting in a composite molecular structure featuring weak, typically van der Waals, bonding between the two components.^32^,^33^ Depending on the details of their microstructure as well as the scientific context of the study, these compounds have also been termed crystallosolvates or polymer-solvent intercalates, clathrates, and complexes.^32^,^33^ Cellulose represents one well-known example of a natural polymer that forms such compounds; synthetic polymer examples include polyoxyethylene,^34^ poly(methyl methacrylate),^29^,^35^ poly(vinylidene fluoride),^36^ as well as isotactic^37^ and syndiotactic polystyrene.^38^–^41^ Despite their unique microstructure, polymer-solvent compounds have, in general, only found relatively limited use as, for example, nanoporous selectively absorbing materials.^42^,^43^ A number of other applications in sensing,^44^,^45^ catalysis,^46^ and packaging^47^ have, however, been proposed recently.

In particular, we present the results of a systematic study of the formation and resulting microstructure of PFO-solvent compounds, investigated by a combination of thermal analysis and X-ray diffraction. We clarify the fundamental role played by the β-phase conformation in compound formation. We further describe how these findings augment the established understanding of PFO solution processing and may allow consequent control of β-phase chain segment fraction within resulting films. While so-called bimolecular crystals/intercalates have been previously reported for mixtures of, for instance, thiophene-based conjugated polymers and fullerene derivatives,^48^,^49^ on the basis of this study PFO represents the first conjugated polymer for which polymer-*solvent* compound formation is clearly demonstrated. We seek, therefore, to also outline new approaches by which the unique molecular-level guest-host crystalline arrangement enabled by compound formation might be exploited to improve the performance of conjugated polymer-based devices.

## EXPERIMENTAL

### Materials

PFO was synthesized using the Suzuki coupling route by the Sumitomo Chemical Company Ltd. The polymer was subjected to extensive purification prior to shipment and was used as received. The weight-average molecular weight, as determined by polystyrene-equivalent gel-permeation chromatography (GPC), was 2.87 × 10^5^ g mol^−1^, with a polydispersity index = 3.0. Decahydronaphthalene (“decalin”) (>98%, mixture of *cis* and *trans* isomers, Acros Organics), toluene (99.7%, anhydrous, Sigma-Aldrich), *n*-dodecane (99%, Acros Organics), 1,2,4-trichlorobenzene (“oTCB”) ( ≥98%, Merck), and *n*-hexadecane (99%, ABCR) were used as received.

### Gel Preparation and Thermal Analysis

Polymer solutions were prepared directly in the standard low-pressure aluminium differential scanning calorimetry (DSC) crucibles. After addition of the required amount of solvent, the crucibles were sealed and carefully weighed before and after measurements to ensure that no solvent loss had occurred. DSC was carried out using a Mettler Toledo DSC 822e instrument that was routinely calibrated using indium standards. As a first step, all mixtures were annealed at temperatures near the boiling point of the solvent for ≥20 min to ensure that homogeneous solutions were obtained. Standard 5 °C min^−1^ heating/cooling rates were used, except for preparation of the so-called “slowly crystallized” gel samples, processed for maximal degrees of crystallinity. For these, the solutions were cooled at 1 °C min^−1^ and then annealed at the corresponding crystallization temperature for 45 min. Melting enthalpy Δ*H*_m_ of free, that is, crystallizable, solvent in these slowly crystallized gels was determined by integrating the corresponding DSC heating thermograms in the −50–20 °C temperature range, which included both the high- and low-temperature endothermal transitions.

### Optical Microscopy

Temperature-dependent optical microscopy was carried out with a Leica DMRX microscope equipped with a Linkam THMS600 hot-stage. Gel samples were sealed between two coverslips, ensuring that no solvent loss occurred.

### Critical-Point Drying of Gels

Dried gels for X-ray diffraction measurements were prepared from the slowly-crystallized as-prepared gels by supercritical drying using a CO_2_ critical-point dryer (SPI Supplies). This method generally allows for interface-free removal of the solvent, thereby preventing the collapse of the swollen as-prepared gel due to the absence of surface tension and resulting in minimal associated changes to the microstructure of the polymer-rich phase.^50^,^51^ The sealed DSC crucibles containing the gel samples were opened and immediately flushed with liquid CO_2_ at ∼15 °C; the samples were then left for 2 h allowing solvent exchange to take place. The temperature was then increased to 37 °C (below the glass transition temperature *T*_g_ of neat PFO), allowing supercritical extraction of CO_2_. Due to the limited miscibility of CO_2_ with the solvents used in this study, the drying process was repeated 3 times to yield maximally solvent-free samples. To ensure data comparability, both as-prepared and dried gel samples were prepared for X-ray diffraction measurements using solutions with identical starting concentrations.

### X-Ray Diffraction

Wide-angle X-ray diffraction (WAXD) was performed on an Oxford Instruments XCalibur PX diffractometer using Mo-Kα radiation (0.71 Å wavelength). Gel, polymer and solvent reference samples were individually sealed inside glass capillary tubes (Hilgenberg; 1.5 mm diameter). Sample temperature was controlled using the Cryojet accessory by streaming temperature-stabilized (±0.1 °C accuracy) nitrogen gas over the capillary tube. The measurements were principally carried out at −100 °C, corresponding to a temperature substantially below the expected compound *T*_g_. While it was not possible to directly measure *T*_g_ for any of the compounds, we estimated the expected *T*_g_ values from the corresponding *T*_m_ values using the empirical equation 1 which holds true for the majority of polymers.^52^


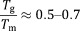
(1)

While the *T*_g_ values determined by this method are very approximate at best, we found that further cooling of the gel samples below −100 °C did not lead to any noticeable changes in the diffraction patterns. The samples were equilibrated at each temperature for 15 min prior to measurement and 20 min integration times were used to record the data. The two-dimensional diffraction patterns were radially integrated following correction for background signal.

## RESULTS

### Thermal Analysis

**FIGURE 1:**
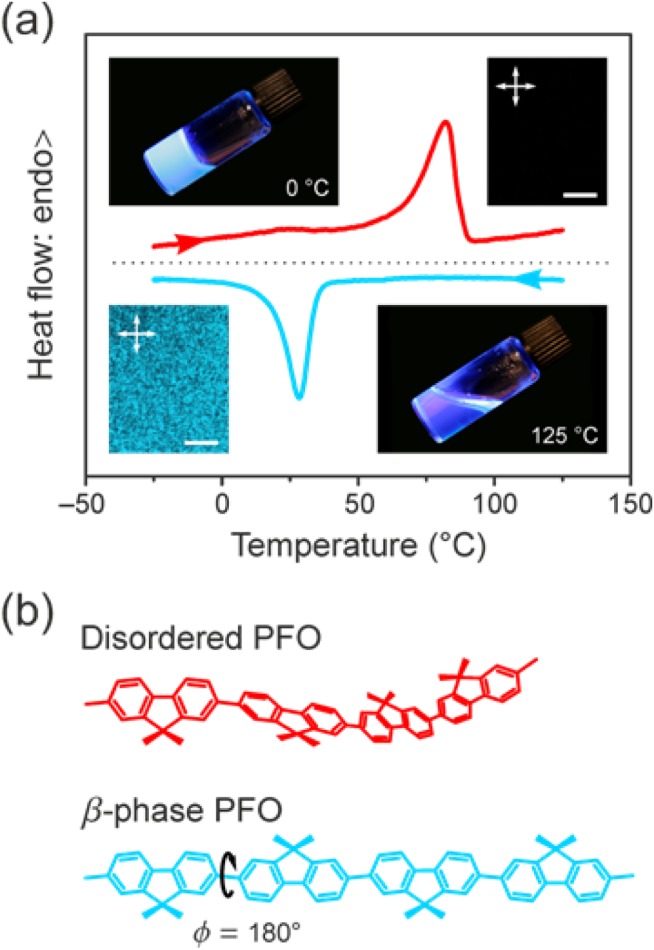
(a) Illustration of the main aspects of the solution-crystallization of PFO, showing, as a specific example, typical DSC thermograms and cross-polarized microscopy images (20 μm scale bars) for PFO–decalin (polymer weight fraction *c*_p_ = 0.3). The dark isotropic image (top right) was taken at 125 °C and the bright scattering image (bottom left) at 0 °C. Photographs of vials containing a (more dilute) *c*_p_ = 0.005 PFO–decalin gel (0 °C, upper left) and solution (125 °C, lower right) under UV illumination are also presented, showing both the thermoreversible solidification and the change in fluorescence color upon gelation. (b) Schematic illustration of the disordered (i.e., wormlike) and β-phase chain conformations of PFO. The octyl (C_8_H_17_) side-chains are omitted for clarity.

Figure 1(a) illustrates common manifestations of solution-crystallization of PFO for the specific case of PFO–decalin mixtures. Also shown in Figure 1(b) are the chemical structure of PFO and schematics of its two principal chain conformations. DSC thermograms [cf. Fig. 1(a)] reveal that the solution-crystallization process is thermoreversible. A crystallization exotherm appears at 28 °C in the cooling trace and a melting endotherm at 82 °C in the heating trace. We note further that the cross-polarized micrograph (bottom left) taken at 0 °C on cooling shows strong crystallite scattering but no evidence for the occurrence of macroscopic liquid–liquid (L–L) demixing and that the corresponding micrograph (top right) taken at 125 °C on heating shows clear dissolution of the semicrystalline microstructure resulting in an isotropic solution.

The inset photographs of vials containing isotropic (bottom right) and crystallized (top left) PFO–decalin mixtures under UV illumination show additionally that solution-crystallization of PFO leads to gelation, that is, formation of a macroscopically coherent “solid” structure, even at polymer weight fractions in solution, *c*_p_, as low as the 0.005 value used for these photographs. The occurrence of macroscopic gelation at such remarkably low polymer content clearly indicates the chain-extended nature of the crystalline polymer structures that are formed as well as a high degree of crystallinity. By comparison, macroscopic gelation of ultra-high molecular weight polyethylene (UHMWPE) in decalin does not occur for the same polymer fraction,^53^ despite the fact that the chain contour length is >23 times higher for UHMWPE relative to the PFO used in this study. Hereafter, for the sake of simplicity, all crystallized PFO solutions will be simply referred to as gels.

The photographs also show a distinct change in fluorescence color between the solution and the gel. While in the solution PFO chains typically adopt a disordered “wormlike” conformation with a broad distribution of intermonomer torsion angles, the gels invariably contain a substantial fraction of PFO chain segments in the so-called β-phase conformation; both chain geometries are schematically illustrated in Figure 1(b). The β-phase conformation refers to a planar-zigzag chain geometry, with a well-defined^10^ inter-monomer torsion angle Φ, which the majority of studies have determined to be Φ = 180°,^4^–^7^,^54^ and a characteristic alignment of the octyl side-chains along the backbone.^10^,^55^ The red-shifted emission from β-phase chain segments (arising from a more extended conformation that supports greater electronic delocalization) typically dominates PL due to efficient excitation energy transfer from the disordered, that is, higher HOMO-LUMO transition energy, parts of the chain ensemble.^4^,^5^,^10^,^19^

**FIGURE 2:**
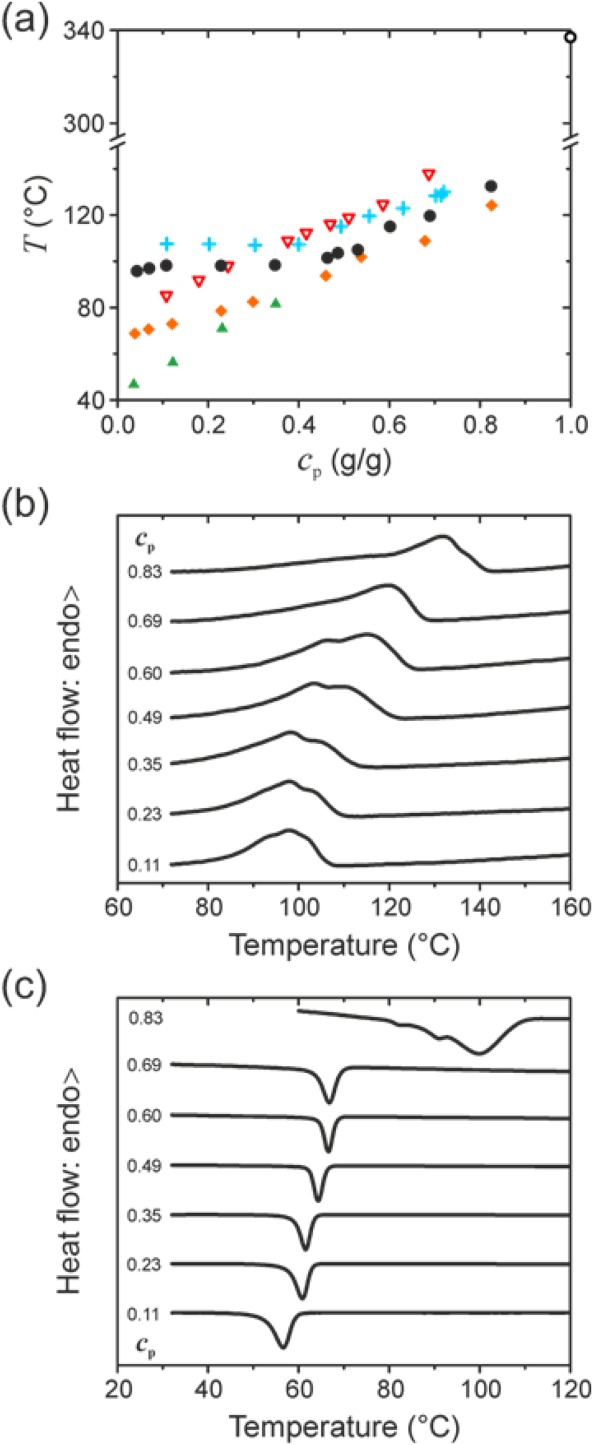
(a) Temperature–composition diagram, representing the variation of peak dissolution temperature *T* with polymer weight fraction *c*_p_ for mixtures of PFO and selected organic solvents. Data is shown for (in order of expected decreasing solvent quality; see Table1) toluene (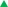), decalin (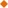), oTCB (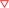), hexadecane (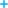) and dodecane (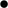). The temperature at which neat PFO undergoes its nematic to isotropic melt transition is also indicated (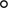, top right corner). For the sake of clarity, dissolution temperature values are reported only for the endotherm with the highest peak heat flow. (b) Heating and (c) cooling DSC thermograms for PFO—dodecane mixtures. The corresponding *c*_p_ values are indicated.

In order to investigate the thermal transitions involved in the crystallization and dissolution of PFO, DSC was performed on mixtures with a range of different organic solvents. [*N.B*. DSC thermograms of neat PFO can be found in *Part II* of this study.] While all previous reports were for relatively dilute (≤ 50 mg mL^−1^) PFO solutions,^30^ in this study the mixtures were prepared (see Experimental) over a much wider concentration range (e.g., 33–3540 mg mL^−1^ for PFO–dodecane). Figure 2(a) presents the temperature–composition diagrams for different PFO-solvent combinations, showing, in each case, the variation of peak dissolution temperature with polymer weight fraction, *c*_p_ (g/g). The reported dissolution temperatures correspond to the peak of the endothermal transitions observed in heating DSC thermograms; when two overlapping endotherms were observed then, for the sake of clarity, the endotherm with the highest peak heat flow was selected for determining the dissolution temperature.

As expected,^56^ addition of solvents that exhibit non-negligible interaction with the polymer leads to dissolution temperatures that are much lower than the thermal transition to the isotropic melt (∼340 °C) observed for neat PFO. The magnitude of this temperature depression, judged from the *c*_p_ <0.2 section of the diagram, generally increases with solvent “quality,” quantified as the squared difference, (Δ*δ*)^2^, between the Hildebrand solubility parameters, *δ*, of PFO and solvent^4^,^57^ (see Table1). We note that the Hildebrand solubility parameters are typically derived from the respective cohesive energy densities^57^ and therefore quantify the overall attractive forces without explicitly separating them into the dispersive, polar and hydrogen bonding contributions.^58^ Nevertheless, since the latter two interactions are likely to be negligible for PFO and the selected solvents, the analysis of their solubilities based on the Hildebrand solubility parameters is deemed to be adequate in the present case.^4^

**Table 1:** Hildebrand solubility parameters, δ, for PFO^4^,^5^ and the solvents^57^ used in our study

Polymer/Solvent	*δ* (cal^1/2^ cm^−3/2^)	(Δ*δ*)^2^ (cal cm^−3^)
PFO	9.2	–
Toluene	8.9	0.09
Decalin	8.7	0.25
oTCB	9.9	0.49
Hexadecane	8.0	1.44
Dodecane	7.8	1.96

(Δ*δ*)^2^ is the squared difference between the solvent solubility parameter and that of PFO. Lower (Δ*δ*)^2^ values correspond to lower heats of mixing and, therefore, to higher solvent “quality”; solvents are listed in quality order from good (top) to poor (bottom).

Also of note are the distinct “kinks,” that is, changes in gradient, seen most clearly for dodecane and hexadecane at *c*_p_ ≈ 0.45. As will be shown below, these are due to the occurrence, typically at high polymer weight fractions, of dynamic recrystallization/melting transitions prior to complete dissolution. [*N.B*. This data will be subsequently revisited (cf. Fig. 6; *vide infra*), when more rigorous, albeit nonequilibrium, temperature–composition “phase” diagrams will be presented.]

Representative heating and cooling DSC thermograms are shown for PFO–dodecane mixtures in Figure 2(b,c), respectively. Crystallization is observed as a single exothermal peak in the cooling thermograms up to *c*_p_ = 0.83, at which point *solution*-crystallization via β-phase chain segment formation then coexists with the standard *melt*-crystallization of neat PFO into an alternative microstructure termed the “α-phase.”^8^ This coexistence results from there being insufficient solvent in the mixture to form a uniform, stoichiometric, polymer-solvent compound (*vide infra*). The process of gel melting/dissolution, as observed in the heating thermograms, is, conversely, somewhat more complicated. For moderately good solvents, that is, toluene, decalin, and 1,2,4-trichlorobenzene (oTCB), gel melting is observed as a single endotherm (see Supporting Information Figs. S1–2). For moderately bad solvents, that is, dodecane (shown here) and hexadecane, two overlapping endotherms are observed in the heating thermograms [cf. Fig. 2(b)]. These were investigated in a control experiment (see Supporting Information Fig. S3), in which a solution was crystallized at a constant cooling rate and re-melted at varied (1–20 °C min^−1^) heating rates. The relative magnitude of the high-temperature endotherm was observed to decrease at higher heating rates, which indicates that only the low-temperature endotherm corresponds to gel melting, whereas the high-temperature endotherm is due to the process of dynamic polymer recrystallization and simultaneous melting.^38^ This assignment is corroborated by the fact that such behavior at low/moderate polymer weight fractions is only observed for the moderately bad solvents, in which the driving force for PFO recrystallization is increased for thermodynamic reasons.

Finally we note that all studied PFO gels exhibited pure β-phase PL, shown in Supporting Information Figure S4, despite the possible coexistence of α-phase crystalline chains for the gels with the highest *c*_p_; this is due to preferential excitation energy transfer to the lower HOMO-LUMO transition energy β-phase chain segments. We also note that the solutions and gels did not exhibit any appreciable green-band (g-band) emission,^59^,^60^ even after repeated heating-cooling DSC cycles followed by compaction and desiccation of the gel, indicating that thermo-oxidative degradation of PFO was minimal (cf. Supporting Information Fig. S4). More detailed optical spectroscopy results will be presented in *Part II* of this study, which specifically focusses on the differences in solution-crystallization behavior for PFO and a related polyfluorene copolymer with a modified side-chain structure. However, for the purposes of this study we can take it as implicit from the PL data that solution-crystallized PFO chains adopt the β-phase conformation. This is also confirmed by the close correspondence between X-ray diffraction patterns recorded for PFO gels (*vide infra*) and the diffraction data reported elsewhere for solution-grown β-phase crystals.^26^,^54^

As already discussed in the introduction, polymer-solvent compounds comprise a structure in which the two components co-crystallize, with solvent molecules incorporated into the periodically arranged cavities created by, for instance, a particular helical conformation of the polymer backbone. Depending on solution concentration, there can additionally be so-called “free” solvent molecules that can undergo the usual crystallization/melting transitions expected for the neat solvent. Crystallizing and re-melting these free-solvent molecules allows for the free-solvent melting enthalpy Δ*H*_m_ to be determined, thus providing additional information on gel composition and structure.^35^,^37^,^61^ Conversely, the solvent molecules involved in the structure of the compound cannot undergo independent thermal transitions and, therefore, do not contribute to the measured Δ*H*_m_.

**FIGURE 3:**
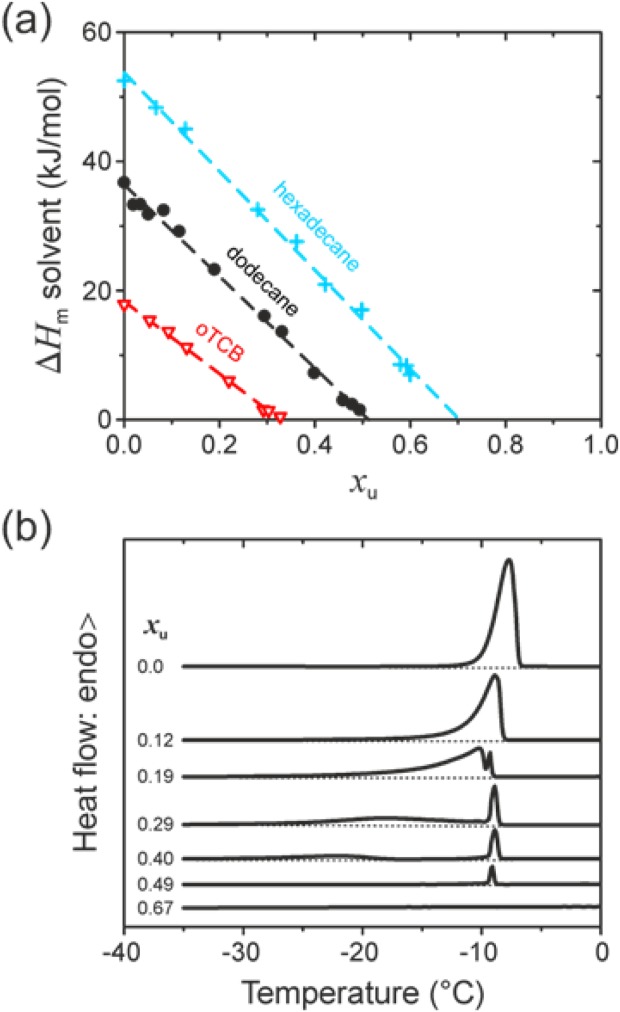
(a) Free-solvent melting enthalpy, Δ*H*_m_, as a function of the PFO repeat unit molar fraction, *x*_u_, for “slowly crystallized” PFO gels with dodecane (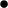), hexadecane (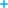) and oTCB (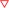). Dashed lines indicate linear fits to the data. (b) Representative DSC heating thermograms (offset for clarity), parametric in *x*_u_, for PFO–dodecane gels; the extrapolated baselines (dotted lines) highlight the presence of additional (weak) endotherms for some of the data.

A DSC study was carried out between −50 and 20 °C (spanning the free-solvent crystallization and melting temperature range for the selected solvents) for slowly crystallized gels, that is, those possessing maximal degrees of crystallinity (see Experimental). Figure 3(a) shows the extracted variation of free-solvent Δ*H*_m_ with gel composition, expressed here as the PFO repeat unit molar fraction *x*_u_, for gels with oTCB, dodecane and hexadecane. Plotting in this way helps to confirm polymer-solvent compound formation^35^,^37^,^61^ and allows the compound stoichiometry to be readily determined.

Gibbs' phase rules dictate that the enthalpy associated with the first-order melting transition of the free-solvent should vary linearly with its concentration. It is indeed observed that increasing the PFO molar fraction, *x*_u_, results in a linear diminution of Δ*H*_m_. Extrapolating the linear fits [dashed lines in Fig. 3(a)] to Δ*H*_m_ = 0 allows the PFO-solvent compound concentration *x*_u_* and, therewith, stoichiometry (the number of solvent molecules per polymer repeat unit, F8) to be estimated; results are summarized in Table2. We note that the reported stoichiometries may be somewhat inaccurate since our analysis assumes full transformation of each solution into polymer-solvent compound plus free-solvent and ignores the presence of any residual dissolved polymer fraction for which a higher degree of solvation can be expected. This situation would tend to overestimate the amount of solvent incorporated within the compound. Reassuringly, however, the deduced stoichiometry values are consistent with the results of X-ray diffraction analysis performed on the same samples (*vide infra*).

**Table 2:** Compound concentrations, *x*_u_*, expressed in terms of the PFO repeat unit molar fraction, and corresponding stoichiometries determined by (free) solvent melting enthalpy measurements

Solvent	*x*_u_*	Stoichiometry (F8:Solvent)	*c*_p_*
Dodecane	0.51 ± 0.01	1:1	0.70
Hexadecane	0.70 ± 0.02	2:1	0.80
oTCB	0.33 ± 0.01	1:2	0.51

To facilitate data comparison, *x*_u_* values are also given as polymer weight fraction, *c*_p_*.

The DSC heating thermograms recorded in the free-solvent melting experiments for PFO–dodecane gels are shown in Figure 3(b). These reveal more complex behavior than is evident simply from the Δ*H*_m_ versus *x*_u_ plots [Fig. 3(a)]. For *x*_u_ = 0 we see a single melting endotherm that peaks at −8 °C as expected for neat dodecane.^62^ For concentrations in the range 0.19 < *x*_u_ < 0.4 two endotherms are seen: one especially sharp peak close to −9 °C and a second feature that broadens, weakens and shifts to lower temperatures as *x*_u_ increases. These observations point to free-solvent being present in the form of both a subset of very well-defined crystals (sharp endotherm) and a second broader distribution of crystals with a range of decreasing sizes which, in accordance with Gibbs' theory, melt at lower temperature than the quasi-infinite crystals.^41^ Finally, for *x*_u_ = 0.67 no free-solvent melting is detectable (flat DSC thermogram) as all of the solvent is trapped within polymer-solvent compounds.

### X-Ray Diffraction

**FIGURE 4:**
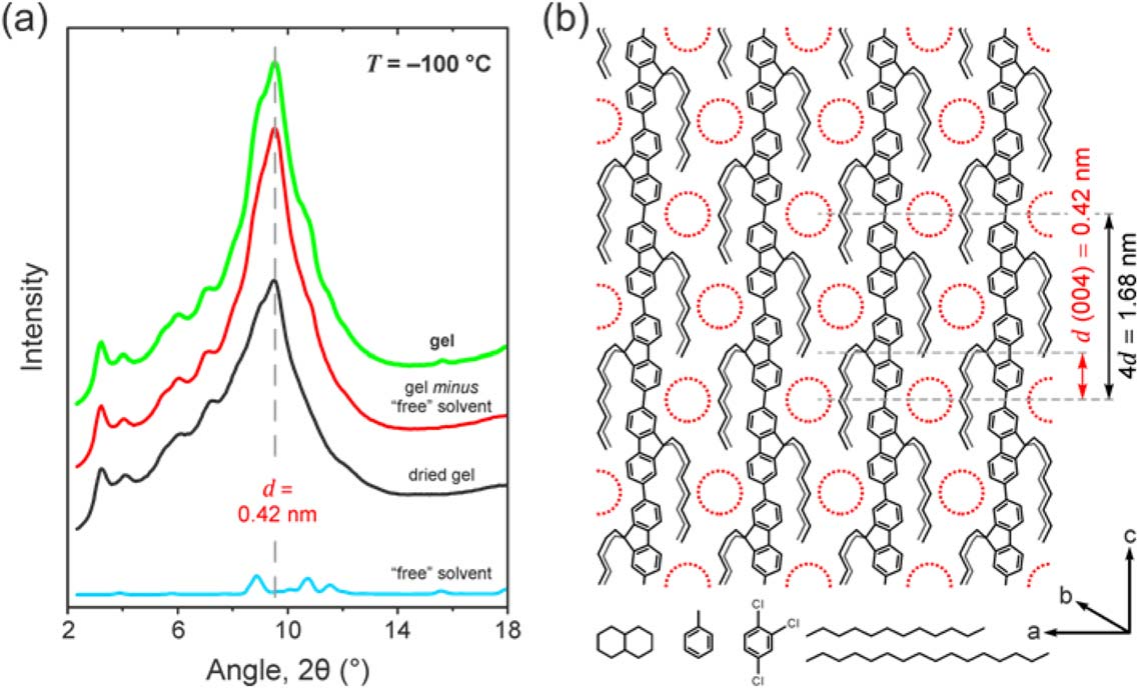
(a) The radially integrated WAXD pattern (green line) recorded for a PFO–hexadecane gel, *x*_u_ ≈ 0.6, at −100 °C (i.e., below the expected glass transition temperature *T*_g_ of the compound and *T*_m_ of the solvent), together with the corresponding normalized diffraction patterns for the dried gel (black line) and the “free” solvent (blue line). Also shown is the diffraction pattern of the gel following subtraction of the normalized diffraction pattern of the free-solvent (‘gel *minus* “free” solvent’; red line). (b) Schematic illustration of the PFO-solvent compound, showing the β-phase chain segments and the cavities that contain the intercalated solvent (red circles). The (004) reflection assigned to the solvent in the compound (*d* = 0.42 nm) as well as the *c*-axis periodicity of intercalated solvent molecules (4*d* = 1.68 nm) are also indicated, with the latter being equivalent to the length of two PFO repeat units in the β-phase conformation (see, e.g., refs. 6,10,54). The chemical structures of the solvents used in this study are shown at the bottom (*left to right*: decalin, toluene, oTCB, dodecane and hexadecane). Note that, while the *n*-alkanes are shown in their all-*trans* conformations, the intercalated solvent molecules are likely to be folded inside the cavities.

WAXD was used to determine the crystalline structure of the PFO-solvent compounds. Slowly-crystallized gel samples were prepared with PFO concentrations, *x*_u_, close to but marginally (∼0.04) below their respective *x*_u_* values in order to simultaneously minimize the presence of α-phase crystalline PFO and free-solvent. The resulting gel diffraction patterns [cf. Fig. 4(a)] recorded at −100 °C nevertheless still comprise contributions from: (i) PFO-solvent compound as well as any residual amorphous PFO fraction and (ii) crystals of the “free,” that is, non-intercalated and crystallizable, solvent. To eliminate the contribution from the latter, we subtracted the normalized diffraction pattern of the neat solvent from that of the as-prepared gel. The reader is directed to Supporting Information Figures S5–7 for further details of WAXD deconvolution and analysis. For comparison, diffraction patterns were also recorded for dried polymer gels obtained by critical-point drying of the as-prepared gels. This approach allows for interface-free removal of the solvent and minimizes the possibility of associated structural changes to the polymer.^51^,^52^ We note that both the as-prepared and dried PFO gels exhibited essentially identical β-phase-dominated PL spectra (not shown) and yielded WAXD patterns with closely corresponding reflection peaks for the polymer (see Supporting Information Fig. S5).

The corresponding set of diffraction patterns for a *x*_u_ ≈ 0.6 PFO–hexadecane gel is shown in Figure 4(a). The pattern for the gel subtracted with the free-solvent contribution [cf. ‘gel *minus* “free” solvent’ data in Fig. 4(a)] features a strong reflection at *d* = 0.42 nm; note that this peak is *unique to the gel* and does not have a counterpart in the diffraction pattern of the free solvent. Equivalent reflections are seen at the same *d*-spacing for PFO gels in dodecane and oTCB, thereby demonstrating the generality of the adopted structure for different solvents and emphasizing the dominant role of the solution-crystallized PFO chains in determining this structure. Furthermore, the same reflection peak is also observed in the diffraction pattern of the *dried* PFO–hexadecane gel, albeit with a reduced intensity in comparison with that of the as-prepared gel [see Fig. 4(a)].

A strong reflection at *d* = 0.42 nm has previously been observed for solution-processed β-phase-rich solid PFO samples^9^,^54^,^64^ and was attributed to a *c*-axis backbone periodicity, corresponding, as it does, to half the length of the PFO repeat unit.^5^ Specifically, Liu et al.^54^ used X-ray and selected-area electron diffraction (SAED) to study the structure of solution-grown β-phase crystals of monodisperse PFO oligomers. It was reported that, following solvent removal, the β-phase crystals comprised an orthorhombic unit cell with its *c*-axis length (3.36 nm) corresponding to the length of four fully-extended fluorene repeat units. Hence, in that study the peak at *d* = 0.42 nm was indexed as the (008) reflection of the β-phase chain segments within these solution-grown crystals.^54^

Since the placement of solvent molecules within the PFO-solvent compound would be dictated by the chain conformation of the polymer, we attribute the peak at *d* = 0.42 nm observed for the as-prepared gels to be *in part* due to the (004) reflection of the intercalated solvent molecules in the compound. This is corroborated by the fact that the *d* = 0.42 nm reflection (i) is more intense for the as-prepared gel compared with that observed for the dried gel [cf. Fig. 4(a)] and (ii) substantially increases in intensity and sharpens upon cooling the gel samples to −100 °C below the expected *T*_g_ of the PFO-solvent compound (see Supporting Information Fig. S7). In this case, 4*d* corresponds to the *c*-axis periodicity of the intercalated solvent which, reassuringly, is equivalent to the length of two PFO repeat units in the β-phase conformation.^6^,^10^ We note that the angular resolution of our instrument prevents us from also observing the (001) reflection peak for the intercalated solvent. An additional reason why the observed reflection is unlikely to be related to solvent placement along the *a*- or *b*-axis is that, due to the different compound stoichiometries and, hence, solvent volumes per cavity, the *d*-spacings along these axes would be expected to vary between the three solvents.^63^ Conversely, due to the rigid chain geometry of the β-phase, the *d*-spacings along the *c*-axis would be identical for different solvents and stoichiometries, as indeed was observed experimentally. Additional WAXD data, notably for a different solvent and two contrasting polymer side-chain structures, is presented in *Part II* of this study.

The proposed structure of the PFO-solvent compound is shown schematically in Figure 4(b). The planar-zigzag chain structure^4^–^7^ and the alignment of the octyl side-chains along the backbone^10^,^55^ are unique to the β-phase conformation. The packing distance of adjacent β-phase chain segments along the *a*-axis is consistent with the interdigitated model proposed previously for β-phase PFO.^24^,^54^,^65^ Taken together, these two structural aspects lead to the formation of cavities [red circles in Fig. 4(b)] which allow for the intercalation of small-molecular solvents of appropriate volume, such as those used in this study (cf. chemical structures in the same figure).

As an additional check, we investigated shrinkage of the polymer unit cell upon critical-point drying of the gels in dodecane, hexadecane and oTCB. The (100) reflection of PFO is well-resolved in all three gels and appears at *d* ≈ 1.26 nm. In the case of a polymer-solvent compound, the amount by which *d* (100) shrinks following solvent removal is expected to be proportional to the volume of solvent per cavity, *V*_c_, in the as-prepared gel. On the other hand, if PFO does not form a compound then the shrinkage of *d* (100) (if any) would not be correlated with *V*_c_.

**FIGURE 5:**
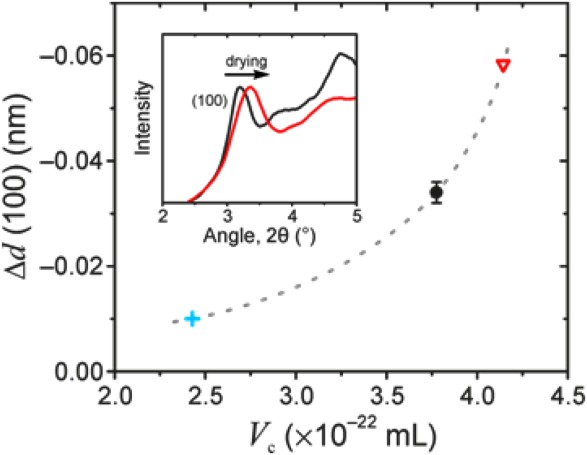
Change in Bragg distance, Δ*d*, of the (100) PFO reflection peak following supercritical drying of PFO gels with dodecane (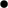), hexadecane (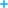) and oTCB (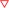), plotted as a function of solvent volume per cavity, *V*_c_, of the polymer-solvent compound. The dashed grey line serves as a guide to the eye. Inset shows representative diffraction patterns of *x*_u_ ≈ 0.3 PFO–oTCB gels in the as-prepared state (black line) and following supercritical extraction of the solvent (red line). The arrow indicates the shift of the (100) reflection. All data were recorded at −100 °C and for polymer concentrations marginally (∼0.04) below the corresponding compound concentrations *x*_u_*.

Figure 5 confirms that the decrease in Bragg distance for the (100) reflection of PFO is proportional to *V*_c_, calculated using the previously determined compound stoichiometries and the known molar volumes for the respective solvents. The inset in Figure 5 shows representative WAXD data for the as-prepared and dried *x*_u_ ≈ 0.3 PFO–oTCB gels. The increase in the width of the (100) reflection following drying can be attributed to (i) decreasing crystal size due to the disruptive effect of solvent removal, and (ii) distortion of the crystal lattice during unit cell shrinkage.

The volume of a single cavity in the PFO-solvent compound [cf. Fig. 4(b)] can be estimated from the maximum obtained *V*_c_ value (two oTCB molecules per cavity) and the volume of hexadecane which was found to be too large for a single cavity (F8 : solvent stoichiometry = 1:2). This yields an estimated cavity volume of 4.1–4.9 × 10^−22^ mL for the PFO-solvent compound. We note that this value shows good agreement with a *back-of-the-envelope* calculation based on the dimensions of the compound structure determined by WAXD, which estimates cavity volume at ∼3 × 10^−22^ mL (see Supporting Information Fig. S8).

### Phase Behavior

**FIGURE 6:**
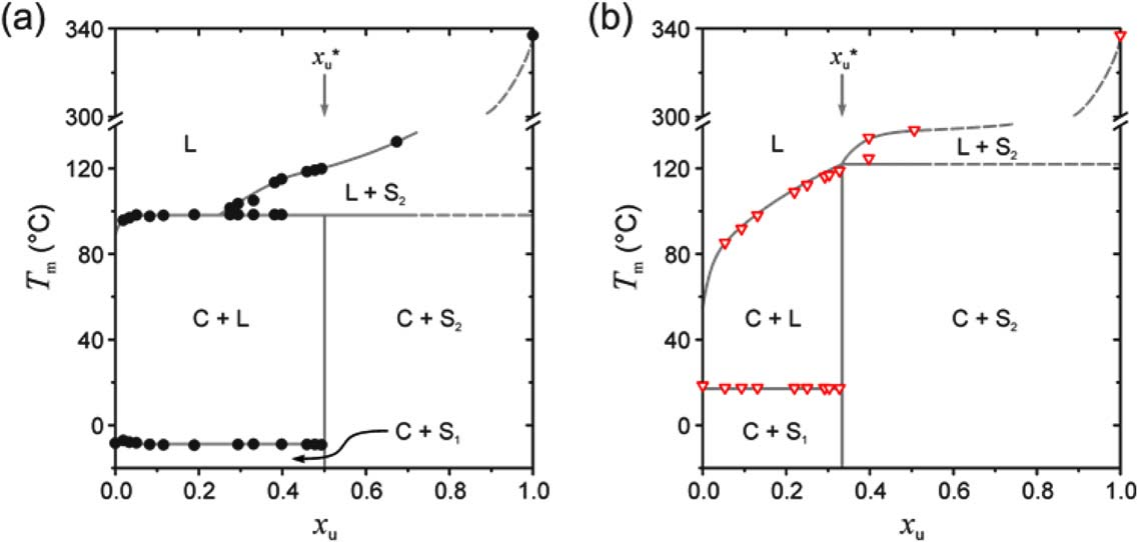
Schematic nonequilibrium temperature–composition phase diagrams for PFO mixtures with (a) dodecane and (b) oTCB. Polymer concentration *x*_u_ is expressed as the F8 repeat unit molar fraction. Symbols represent melting temperatures; solid lines are guides to the eye for the observed transitions while dashed lines indicate their probable extensions. The phases are represented as follows: L, liquid solution or melt; S, (semi-)crystalline solid; C, compound; subscripts 1 and 2 refer to the solvent and polymer components, respectively. Compound concentrations *x*_u_* are indicated by the arrows.

Having confirmed the formation of polymer-solvent compounds with PFO by both thermal analysis and WAXD, we now present selected data from Figure 2(a) in the context of nonequilibrium temperature–composition “phase” diagrams, shown in Figure 6. The temperature–composition phase diagram for PFO–dodecane [cf. Fig. 6(a)] is characteristic of an incongruently-melting compound, whereby the compound C transforms into another phase (in this case a liquid plus, most likely, semicrystalline polymer (L + S_2_)) at concentrations below *x*_u_* prior to its complete melting.^33^ Such behavior has been observed for syndiotactic polystyrene (sPS) gels in *trans*-decalin, for which a compound with stoichiometry 1:1 has been demonstrated.^41^ Conversely, the temperature–composition phase diagram for PFO–oTCB [cf. Fig. 6(b)] is typical of a singular-melting compound, for which direct melting occurs, with maximum melting temperature found at the compound concentration *x*_u_*.^33^ This type of compound has been found for sPS gels in tetralin^41^ and toluene,^33^ both featuring 1:1 stoichiometry. Typically, incongruently-melting compounds are observed when poorer solvents are used^39^ which is indeed the case for PFO, considering the less-matched solubility parameter of dodecane compared with oTCB (cf. Table1). Interestingly, the ∼25 °C difference in maximum *T*_m_ of the two compounds shown in Figure 6 suggests that the stability of the compound is higher for PFO–oTCB. One possibility is that this is due to a higher volume of solvent per cavity in the compound (4.1 × 10^−22^ mL compared with 3.8 × 10^−22^ mL for dodecane) which results in stronger van der Waals bonding between the polymer and intercalated solvent. An alternative explanation is that the stability of the PFO compound is proportional to the solvent quality; this correlation has been proposed for sPS compounds, although without considering the possible stoichiometric differences.^39^

On a cautionary note, we should emphasize that the presented phase diagrams can serve only as an approximation to the equilibrium phase behavior. First, although PFO gels were dealt with as two-component systems, strictly speaking the polymer can hardly be treated as a single component given its large polydispersity and incomplete formation of the β-phase conformation. Second, since the gels were prepared by dynamic crystallization at finite cooling rates, their reported melting temperatures do not represent equilibrium values. Nevertheless, as shown in previous reports,^40^,^66^ these nonequilibrium temperature-composition phase diagrams provide an adequate qualitative indication of the phase behavior.

## DISCUSSION

Our demonstration of PFO-solvent compound formation invites discussion, as well as further research, in two specific directions:

- These observations substantially add to our understanding of the β-phase conformation, and in particular show that, due to its planar-zigzag geometry, this conformation allows for intercalation of solvent into the on-chain cavities, thereby stabilizing the backbone. In this respect, the situation is similar to sPS-solvent compounds that feature chains in the helical, so-called δ-phase, conformation. The δ-phase of sPS can be formed via crystallization from solutions as well as by exposure of amorphous or crystalline sPS films to an appropriate solvent in liquid or vapor form.^32^ The latter methods, namely immersion in solvent–nonsolvent mixtures and solvent vapor annealing, are also commonly used to introduce the β-phase conformation in solid-state PFO.^6^,^12^,^17^,^18^ Approaching β-phase chain segment formation via crystallization protocols may stimulate new approaches to fabrication of solid-state PFO samples with β-phase content greater than the ∼45% achieved to date.^11^ Confirmation of the fundamental role played by solvent in formation of the β-phase and first elucidation of the stabilizing effect of polymer-solvent compound formation encourages further studies of solvent and processing temperature optimization. Our preliminary results indicate that the solution-crystallization rate of PFO is reduced in low molar volume solvents (see Supporting Information Fig. S9), adding a new parameter axis to solvent selection. Such considerations can be especially important for fabrication when PFO films maximally free from β-phase chain segments are required.^3^,^18^
  On a separate note, it should be mentioned that the first observations of the β-phase conformation in PFO were made following thermal postprocessing of glassy spin-coated PFO films via *in vacuo* cooling to and reheating from −196 °C.^4^–^6^ Given the seemingly solvent-free formation of the β-phase in that case, as well as other observations of β-phase formation during, for instance, solution-casting on a water surface,^67^,^68^ it would be most interesting to further study the differences between these and more common solvent-based approaches to inducing β-phase in solid-state PFO.
- The formation of a polymer-solvent compound is a molecular recognition process, for which the critical solvent properties are: (a) appropriate volume and, to a lesser extent, (b) matching solubility parameter. Hence, molecular PFO compounds might more generally be formed with the organic solvent replaced by a small molecule of desired optoelectronic properties, thereby allowing the fabrication of ultra-regular molecular-level blends comprising a PFO host and a small-molecular guest. A judicious choice of guest molecule might therefore enable improved device performance for PFO compounds in terms of, for example, charge–carrier injection and transport, energy transfer, charge separation, and stability against photo- or thermal-degradation. This is expected to prove an interesting avenue for future research.

## CONCLUSIONS

Polymer-solvent compound formation has been demonstrated for PFO and a range of organic solvents, observed as solution-crystallization and accompanied by thermoreversible gelation. The crystalline structures in the gels consist of solvated β-phase crystals, in which solvent molecules are intercalated into the on-chain cavities characteristic of the PFO β-phase chain conformation. The properties (such as volume and solubility parameter) of the small-molecular species were found to play a key role in compound formation, significantly affecting the stoichiometry of the resulting compound, as well as its stability. Comparisons have been drawn between the compound formation for PFO and similar phase behavior in other systems, notably those based on syndiotactic polystyrene. Our findings clarify the nature of the β-phase conformation and suggest new strategies for controllable solution-processing of PFO.

Having shown compound formation with simple molecules like 1,2,4-trichlorobenzene and dodecane, future work will extend this approach to other guest molecules with similar physicochemical properties (e.g., molecular volume) with the aim of engineering new functionalities in solid-state PFO-based blends.
