# Supplementary material for: Solution-Crystallization and Related Phenomena in 9,9-Dialkyl-Fluorene Polymers. I. Crystalline Polymer-Solvent Compound Formation for Poly(9,9-dioctylfluorene)
Source: J Polym Sci B Polym Phys. 2015 Aug 19;53(21):1481–91. doi: 10.1002/polb.23798 (PMC4584509; doi:10.1002/polb.23798)
Supplement: Supplementary file 1 — Supplementary Information [file polb0053-1481-sd1.pdf]

Supporting Information

"Solution-Crystallization and Related Phenomena in 9,9-Dialkyl-Fluorene Polymers. I. Crystalline Polymer-Solvent Compound Formation for Poly(9,9-dioctylfluorene)"

Aleksandr Perevedentsev e*t al.*

1. DSC Thermograms for PFO in Decalin and oTCB.

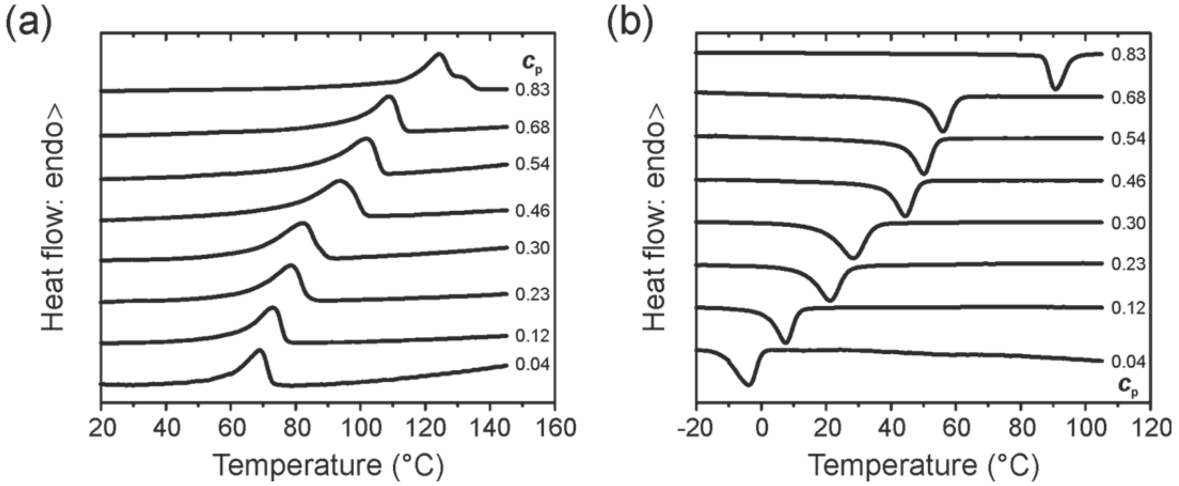

FIGURE S1 **(a)** Heating and **(b)** cooling DSC thermograms recorded for PFO–decalin mixtures with the corresponding polymer weight fraction, c_p, values indicated. Standard 5 °C min^{-1} rates were used.

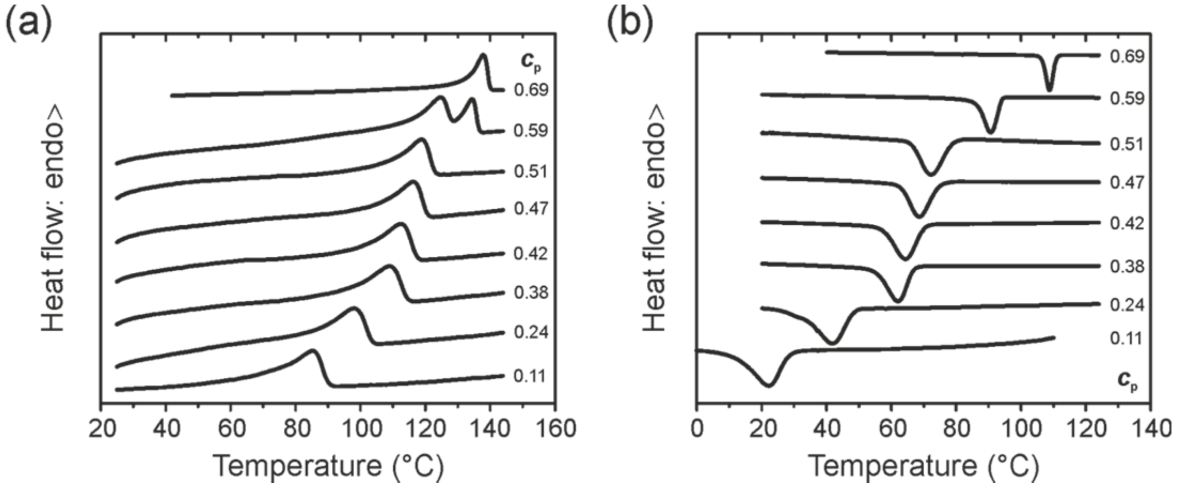

FIGURE S2 **(a)** Heating and **(b)** cooling DSC thermograms recorded for PFO–oTCB mixtures with the corresponding c_p values indicated. Standard 5 °C min^{-1} rates were used.

Note that for both of these systems, melting/dissolution and crystallization/gelation are observed as *single* endo- and exo-thermic events, respectively, up to high c_p where more complicated behaviour is observed due to solvent deficiency for the formation of a stoichiometric compound. This is attributed to dynamic melting and recrystallization, as well as the likely coexistence within the gels of PFO chains crystallized in the non-solvated melt-crystalline-type α-phase.[1,2]

2. Dynamic Recrystallization and Melting in PFO.

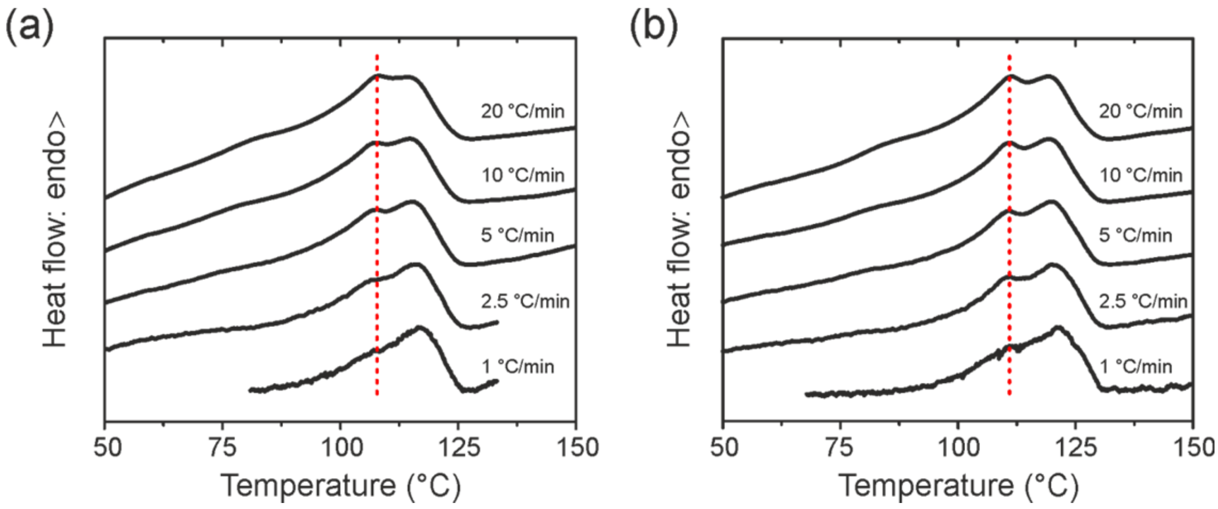

FIGURE S3 Heating rate dependence of the DSC thermograms recorded for PFO gels in **(a)** dodecane and **(b)** hexadecane, with polymer concentrations c_p = 60 and 56 wt % respectively. Mixtures were crystallized at constant 2 °C min^{-1} rates and re-heated at the indicated rates. Dashed red lines are a guide to the eye for the position of the low-temperature endotherms.

The relative contribution of the high-temperature endotherm is reduced when higher heating rates are used. This is understood to be due to kinetically-hindered recrystallization at high heating rates which in turn results in lower relative enthalpy of the high-temperature endotherm. Such behaviour is well-documented for sPS gels[3] as well as neat sPS.[4]

3. PL Spectroscopy of PFO Gels Prepared by DSC.

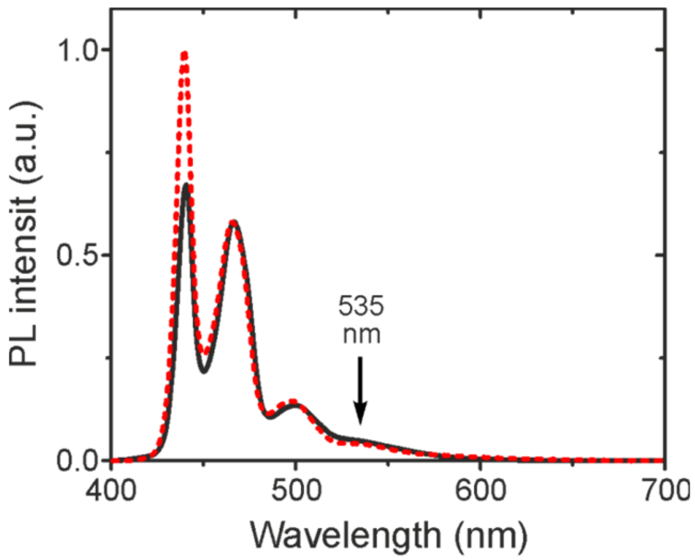

FIGURE S4 Photoluminescence (PL) spectrum of a slowly-crystallized c_p = 0.3 PFO-dodecane gel (solid black line) that has previously undergone repeated heating/cooling cycles in the DSC between –50 and 160 °C. Prior to PL measurement, the gel was pressed into a film at room-temperature using a bench-top hydraulic press (load = 10 tons) and subsequently desiccated to ensure that minimal amount of solvent remained in the film. Also shown is the PL spectrum of a drop-cast PFO thin film (dashed red line) for which the fraction of β-phase chain segments was estimated to be ~30%. Excitation wavelength λ_{ex} = 390 nm in both cases.

The two spectra are normalized by their S_1-S_0 0-1 vibronic in order to correct for the effect of different degrees of self-absorption for the two films. Two features deserve particular mention:

(i) The PL spectrum of the gel is typical of PFO samples possessing a high fraction of chains segments in the β-phase conformation, with S_1-S_0 vibronic peaks located at 440, 467 and 500 nm.

(ii) PL intensity at 535 nm (indicated by the arrow) is comparable for both samples. For PFO, 535 nm is expected to be the spectral position of the peak in green emission due to oxidative degradation of 9,9-dioctylfluorene to 9-fluorenone moieties.[5,6] This indicates that the solution-crystallization behaviour of PFO investigated in this study is unlikely to be complicated by the additional presence of on-chain fluorenone defects.

4. Wide-Angle X-Ray Diffraction (WAXD) Analysis.

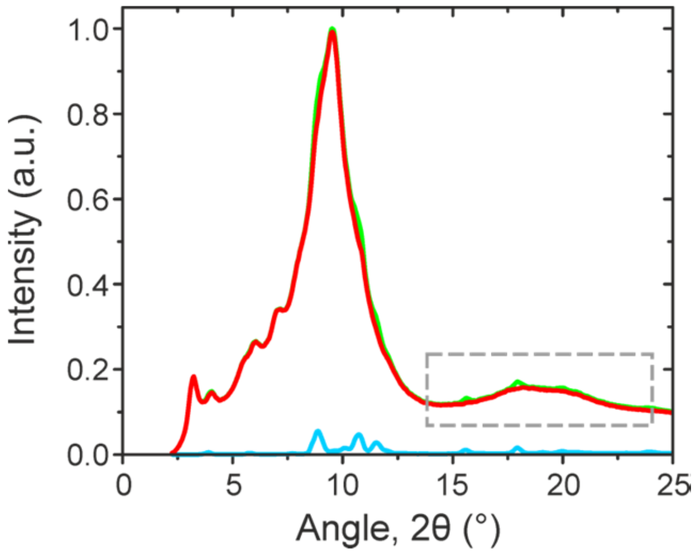

FIGURE S5 Illustration of the deconvolution method applied to WAXD patterns recorded for PFO gels in order to eliminate the contribution of the free-solvent; representative data is shown for $x_u \approx 0.6$ PFO–hexadecane gel at –100 °C. The diffraction pattern of the "free" (i.e. crystallisable) solvent (blue line) is normalized and subtracted from the diffraction pattern of the as-prepared gel (green line); the difference pattern is shown (red line). The dashed rectangle indicates the region preferentially used for normalising the diffraction patterns.

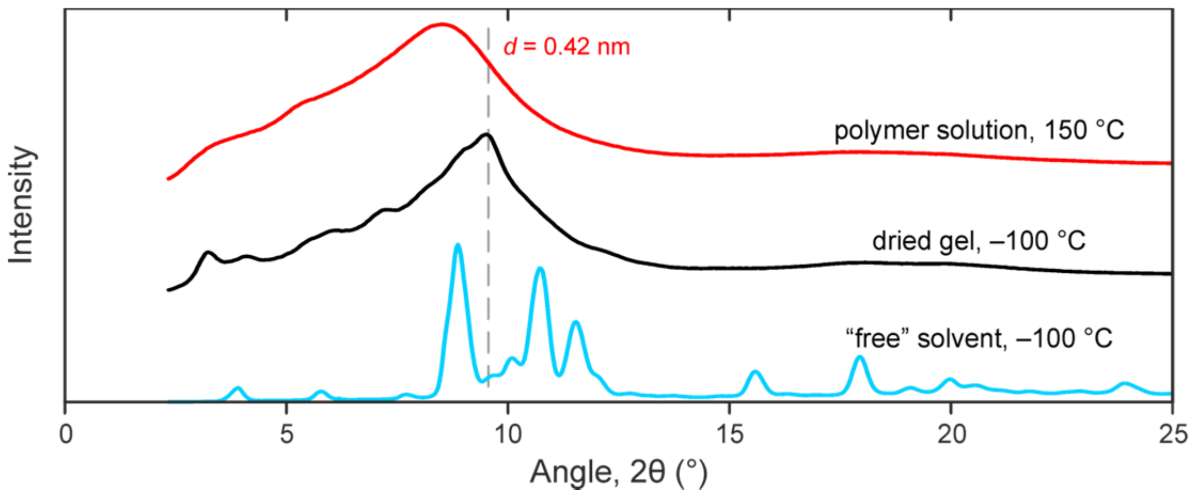

FIGURE S6 Peak-normalized WAXD patterns recorded for x_u ≈ 0.6 PFO–hexadecane: polymer solution at 150 °C (above the dissolution temperature; red line), as well as dried gel (black line) and the neat solvent reference (blue line) at –100 °C. The dashed red line indicates the *d*-spacing (d = 0.42 nm) corresponding to the *c*-axis periodicity of solvent in the compound (see main text and Figure 4 for details).

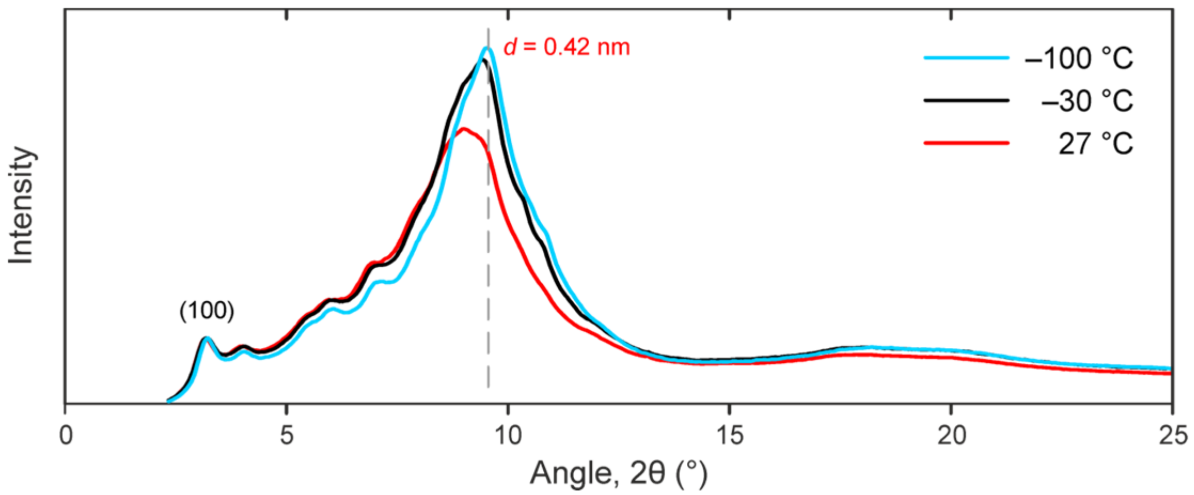

FIGURE S7 WAXD patterns recorded for x_u ≈ 0.6 PFO–hexadecane gels at different temperatures: 27 °C (red line), –30 °C (black line) and –100 °C (blue line). The diffraction patterns are normalised by the (100) reflection for PFO at 2θ ≈ 3.2° (indicated in the figure). The free-solvent contribution has been subtracted in each case (cf. Figure S5). The increase in intensity and sharpening of the reflection at d = 0.42 nm with reducing temperature is evident.

5. Estimating the cavity volume in the PFO–solvent compound from WAXD data.

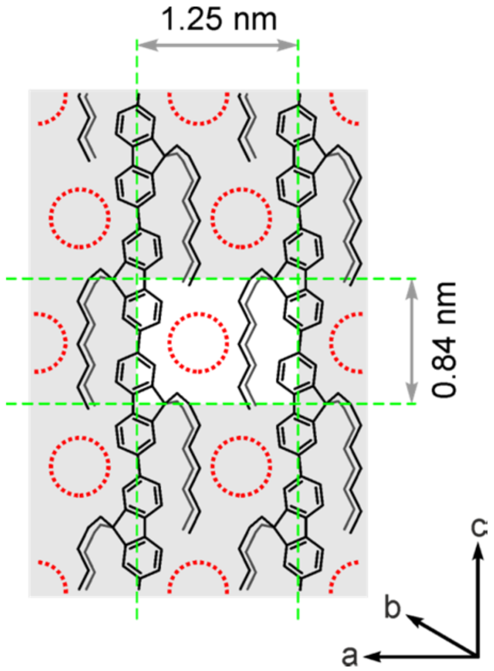

FIGURE S8 Schematic illustration of chain structure in a PFO–solvent compound (cf. Figure 4(b) in the main text).

The maximum length of a single cavity along the *c*-axis corresponds to 0.84 nm, i.e. the length of a F8 repeat unit.[7,8] The spacing of adjacent PFO chains along the *a*-axis was calculated from the (100) reflection in WAXD patterns recorded for as-prepared gels in dodecane, for which the stoichiometry of 1 : 1 was measured. As is evident from Figure S6, only a fraction of the length along the *a*-axis would be available for the placement of intercalated solvent due to the presence of the dioctyl side-chains; thus, for the estimation of cavity volume V_c, it was arbitrarily scaled by 75%. The spacing of adjacent chains along the *b*-axis is estimated as 0.4 nm, calculated from the (020) reflection in the WAXD patterns recorded for the as-prepared PFO–dodecane gels. We note that these values closely correspond to the dimensions of a solvent-free β-phase crystal reported by Liu *et al.*[8] Calculating V_c using these values yields:

$$V_c = (0.75 \times 1.25) \times 0.4 \times 0.84\ \mathrm{nm}^3 = 3.2\ \times 10^{-22}\ \mathrm{mL}$$

The value of ~3 × 10^{-22} mL shows reasonable agreement with $V_c \approx 4.1$–4.9×10^{-22} mL, estimated using the calculated compound stoichiometries and molar volumes of the respective solvents.

6. Non-isothermal crystallization half-times of PFO solutions.

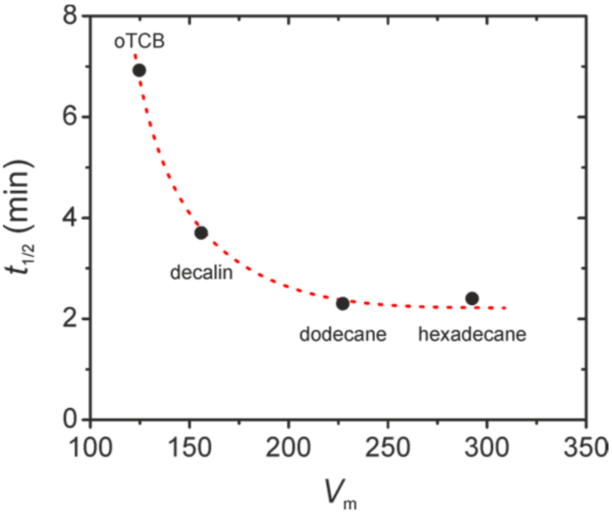

FIGURE S9 Non-isothermal crystallization half-times $t_{1/2}$ measured by DSC for PFO solutions ($x_u \approx 0.05 \pm 0.01$) in solvents with different molar volumes V_m. Constant 1 °C min^{-1} cooling rates were used.

Crystallization half-time is defined as the time elapsed from the onset of crystallization to the system reaching 50% degree of transformation, i.e. the value of relative crystallinity = 50%. Thus, crystallization half-time is *inversely* proportional to crystallization rate. These preliminary results emphasise that judicious choice of solvent is necessary for controllable solution-processing for PFO, e.g. in situations when it is desirable to avoid the presence of β-phase chain segments in the solid-state.

7. References

[1] S. H. Chen, A. C. Su, C. H. Su, S. A. Chen, *Macromolecules* **2005**, *38*, 379.

[2] S. H. Chen, A. C. Su, A. A. Chen, *J. Phys. Chem. B* **2005**, *109*, 10067.

[3] F. Deberdt, H. Berghmans, *Polymer* **1993**, *34*, 2192.

[4] J. Arnauts, H. Berghmans, *Polym. Commun*. **1990**, *31*, 343.

[5] M. Sims, D. D. C. Bradley, M. Ariu, M. Koeberg, A. Asimakis, M. Grell, D. G. Lidzey, *Adv. Funct. Mater.* **2004**, *14*, 765.

[6] E. J. W. List, R. Guentner, P. S. de Freitas, U. Scherf, *Adv. Mater.* **2002**, *14*, 374.

[7] M. Grell, D. D. C. Bradley, X. Long, T. Chamberlain, M. Inbasekaran, E. P. Woo, M. Soliman, *Acta Polym*. **1998**, *49*, 439.

[8] C. Liu, Q. Wang, H. Tian, J. Liu, Y. Geng, D. Yan, *Macromolecules* **2013**, *46*, 3025.
